# Synthesis and biological evaluation of new 1,3-thiazolidine-4-one derivatives of nitro-l-arginine methyl ester

**DOI:** 10.1186/s13065-016-0151-6

**Published:** 2016-02-04

**Authors:** Andreea-Teodora Pânzariu, Maria Apotrosoaei, Ioana Mirela Vasincu, Maria Drăgan, Sandra Constantin, Frédéric Buron, Sylvain Routier, Lenuta Profire, Cristina Tuchilus

**Affiliations:** Department of Pharmaceutical Chemistry, Faculty of Pharmacy, University of Medicine and Pharmacy “Grigore T. Popa”, 16 University Street, 700115 Iasi, Romania; Institut de Chimie Organique et Analytique - ICOA UMR7311, Pôle de chimie, Rue de Chartres, 45100 Orléans, France; Department of Microbiology, Faculty of Pharmacy, University of Medicine and Pharmacy “Grigore T. Popa”, 16 University Street, 700115 Iasi, Romania

**Keywords:** Nitro-l-arginine methyl ester, 1,3-Thiazolidine-4-one, Spectral methods, Antioxidant effects, Antibacterial/antifungal activity

## Abstract

**Background:**

l-Arginine is a semi-essential aminoacid with important role in regulation of physiological processes in humans. It serves as precursor for the synthesis of proteins and is also substrate for different enzymes such as nitric oxide synthase. This amino-acid act as free radical scavenger, inhibits the activity of pro-oxidant enzymes and thus acts as an antioxidant and has also bactericidal effect against a broad spectrum of bacteria.

**Results:**

New thiazolidine-4-one derivatives of nitro-l-arginine methyl ester (NO_2_-Arg-OMe) have been synthesized and biologically evaluated in terms of antioxidant and antibacterial/antifungal activity. The structures of the synthesized compounds were confirmed by ^1^H, ^13^C NMR, Mass and IR spectral data. The antioxidant potential was investigated using in vitro methods based on ferric/phosphomolybdenum reducing antioxidant power and DPPH/ABTS radical scavenging assay. The antibacterial effect was investigated against Gram positive (*Staphylococcus aureus* ATCC 25923, *Sarcina lutea* ATCC 9341) and Gram negative (*Escherichia coli* ATCC 25922, *Pseudomonas aeruginosa* ATCC 27853) bacterial strains. The antifungal activity was also investigated against *Candida* spp. (*Candida albicans* ATCC 10231, *Candida glabrata* ATCC MYA 2950, *Candida parapsilosis* ATCC 22019).

**Conclusions:**

Synthesized compounds showed a good antioxidant activity in comparison with the NO_2_-Arg-OMe. The antimicrobial results support the selectivity of tested compounds especially on *P. aeruginosa* as bacterial strain and *C. parapsilosis* as fungal strain. The most proper compounds were **6g** (R = 3-OCH_3_) and **6h** (R = 2-OCH_3_) which showed a high free radical (DPPH, ABTS) scavenging ability and **6j** (R = 2-NO_2_) that was the most active on both bacterial and fungal strains and also it showed the highest ABTS radical scavenging ability.

**Graphical abstract:**
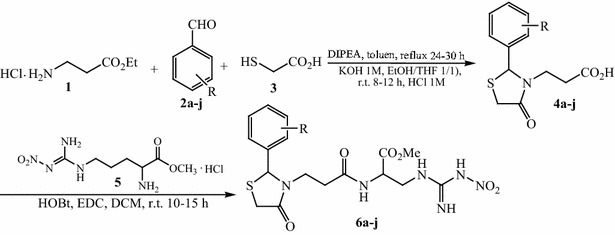
1: ethyl 3-aminopropionate hydrochloride, **2a**–**j**: aromatic aldehydes, 3: thioglycolic acid, **4a**–**j**: thiazolidine-propionic acid derivatives , 5: N_ω_-nitro-L-arginine methyl ester hydrochloride, **6a**–**j**: thiazolidine-propionyl-nitro-L-arginine methyl ester derivatives

## Background

l-Arginine is an amino acid with the highest nitrogen content known for its important role in regulation of physiological processes in humans [1]. This amino acid is considered a semi-essential amino acid because normal cells can not only synthesize arginine *de novo* through the ornithine cycle but also uptake extracellular arginine [2]. It serves as a precursor for the synthesis of proteins and it is also substrate for different enzymes. For example nitric oxide synthase (NOS) converts arginine to nitric oxide (NO) and citrulline. Three isoforms of NOS have been described: endothelial NOS (eNOS), neuronal NOS (nNOS), that are constitutive isoforms (cNOS) and inducible NOS (iNOS) [3]. NO, is an important signal molecule, involved in immune responses, angiogenesis, epithelialization and formation of granulation tissue, vasodilatation of smooth muscle and inhibition of platelets activation/aggregation [4, 5]. The cNOS produce NO in picomolar amounts for short time, being responsible for regulation of arterial blood pressure, while iNOS produces large amounts of NO through cell activation under inflammatory conditions, appearing to be involved in pathophysiological phenomena [3]. Nitro-l-arginine methyl ester (NO_2_-Arg-OMe, L-NAME) is known as selective inhibitor of inducible NOS, which showed antinociceptive effects in mice and reversed thermal hyperalgesia in rats with carrageenan arthritis [6]. It was also reported that L-NAME attenuates the withdrawal from cocaine [7] and prevents the behaviour effects indused by phencyclidin, a dissociative drug [8].

l-Arginine is reported also to act as free radical scavenger, inhibits the activity of pro-oxidant enzymes and thus acts as an antioxidant [9, 10]. This endogenous molecule has also bactericidal effect against a broad spectrum of bacteria, by nitrosation of cysteine and tyrosine residues, which lead to dysfunction of bacterial proteins. This effect could be useful in different conditions as wounds when infection could delay the healing process. The two most common bacteria in wounds are *Pseudomonas aeruginosa* and *Staphylococcus aureus* [11]. In addition, to its role as precursor of NO, l-arginine can be metabolized by arginase to ornithine and urea. Ornithine is an essential precursor for collagen and polyamines synthesis, both required for wound healing processes [12]. Based on all these aspects there has been reported that l-arginine has important roles in Alzheimer disease [13], inflammatory process [14], healing and tissue regeneration [14–16] and also it showed anti-atherosclerotic activity [17, 18].

On other hand the heterocyclic compounds are an integral part in organic chemistry field and constitute a modern research field that is being currently pursued by many research teams [19]. Diversity in the biological response of 1,3-thiazolidine-4-one derivatives had attracted the attention of many researchers for a thorough exploration of their biological potential. These compounds have been reported for their antioxidant [20–22], anti-inflammatory [23], antibacterial/antifungal [24–26], antitumor [27], antidiabetic [28], antihyperlipidemic [29] and antiarthritic [30] effects.

In order to improve the biological effects of l-arginine and, new 1,3-thiazolidine-4-one derivatives have been synthesized. The spectral data (FT-IR, ^1^H-NMR, ^13^C-NMR, MS) of each compound were recorded and the compounds were screened for their in vitro antioxidant potential and antibacterial/antifungal activity.

## Results and discussion

### Chemistry

**Scheme 1:**
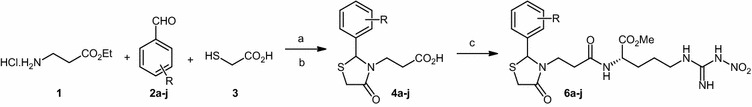
Synthesis of compounds **6a**–**j**. Reagents and conditions: **a** DIPEA, toluene, reflux 24–30 h; **b** KOH 1 M, EtOH/THF (1/1), r.t. 8–12 h then HCl 1 M; **c** *N*_*ω*_-nitro-l-arginine methyl ester hydrochloride (**5**), HOBt, EDC, DCM, r.t. 10–15 h

The synthesis of thiazolidine-4-one compounds derived from L-NO_2_-Arg-OMe was performed in two steps and is summarized in Scheme 1 and Table 1. The first step consisted in formation of the 1,3-thiazolidin-4-one cycle via a *one*-*pot* condensation/cyclization reaction which implies the using of ethyl 3-aminopropionate hydrochloride **1**, different substituted aromatic aldehydes **2a**–**j** and thioglycolic acid **3** using a similar approach described in our previous work [27]. The product of this reaction was treated with KOH to give compounds **4** in satisfactory to very good overall yields. In the second and last step, the formation of amide bond between acid derivatives **4** and *N*_ω_-nitro-l-arginine methyl ester hydrochloride **5** was carried out using classical conditions in presence of 1-ethyl-3-(3-dimethylaminopropyl)carbodiimide hydrochloride (EDC) and 1-hydroxybenzotriazole (HOBt) to lead to new thiazolidine-4-one derivatives with arginine moiety **6a**–**j**.

**Table 1:** Synthesis of derivatives **4** and **6** 
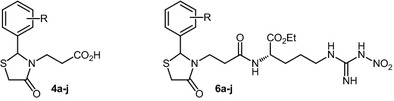

Entry	Comp.	R	**4**, Yield^a^ (%)	**6**, Yield^a^ (%)
1	**a**	H	73	93
2	**b**	4-CH_3_	55	91
3	**c**	4-Cl	59	89
4	**d**	4-F	67	75
5	**e**	4-Br	78	87
6	**f**	4-OCH_3_	55	86
7	**g**	3-OCH_3_	57	78
8	**h**	2-OCH_3_	64	76
9	**i**	3-NO_2_	63	50
10	**j**	2-NO_2_	82	91

^a^Yields are indicated in isolated compounds

The structure of the compounds was assigned on the basis of spectral data (IR, ^1^H-NMR, ^13^C-NMR, MS) which are provided in the Experimental Section. The spectral data for compounds **4a**–**j** were presented in our previous paper [31].

The analysis of IR spectral data obtained for compounds **6a**–**j** showed that the NH group corresponding to the amide bond formed was identified between 3305 and 3294 cm^−1^ in the form of a medium or low intensity bands. The specific anti-symmetric valence vibration of CH_2_ group has been reported in the range of 2940–2825 cm^−1^ and overlaps with specific absorption band of CH group, which is identified in the same range. The C=O group was identified as three absorption bands: the absorption band in the 1760–1670 cm^−1^ corresponds to ester group (COOCH_3_), in the area of 1686–1647 cm^−1^ was identified the absorption band corresponding to C=O from amide bond and the group C=O from the thiazolidine-4-one moiety appears in the range of 1647–1610 cm^−1^. The vibration of C–S bond, specific for thiazolidine-4-one, was identified between 694 and 668 cm^−1^.

The formation of **6a**–**j** has also been proved by the NMR data. The thiazolidine-4-one structure was proved by characteristic proton signals. The proton of S–CH–N group appears as doublet in the range of 5.72–6.08 while the two protons from thio-methylene group (S–CH_2_) were recorded dispersed; the first resonates between 4.41 and 4.72 ppm, and the second between 3.80 and 4.07 ppm. The amide bond (–NH–CO) was proved by the characteristic proton signal which resonates as singlet in the range 8.48–8.68 ppm.

In the ^13^C-NMR spectra the carbons of thiazolidine-4-one system appear between 64.36 and 62.65 ppm for S–CH–N and between 34.53 and 33.10 ppm for –CH_2_–S. The signals for the three CO groups (CO_thiazolidine_, CO_amide_, CO_ester_) appear in the range of 173.24–160.39 ppm, which confirm the success of peptide coupling reaction.

The proton and carbon signals for other characteristics groups were observed according to the expected chemical shift and integral values. The NMR spectral data coupled with mass spectra strong support the proposed structures of each synthesized compounds.

### Biological evaluation

#### Antioxidant activity

The antioxidant activity was evaluated using in vitro tests: DPPH and ABTS radical scavenging, phosphomolydenum reducing antioxidant power and ferric reducing antioxidant power assays. For each compound it was calculated effective concentration 50 (EC_50_) by linear regression. The results were expressed as EC_50_ value which represents the concentration where half of the substrate is being reduced by the tested compounds.

#### The DPPH radical scavenging assay

**Fig. 1:**
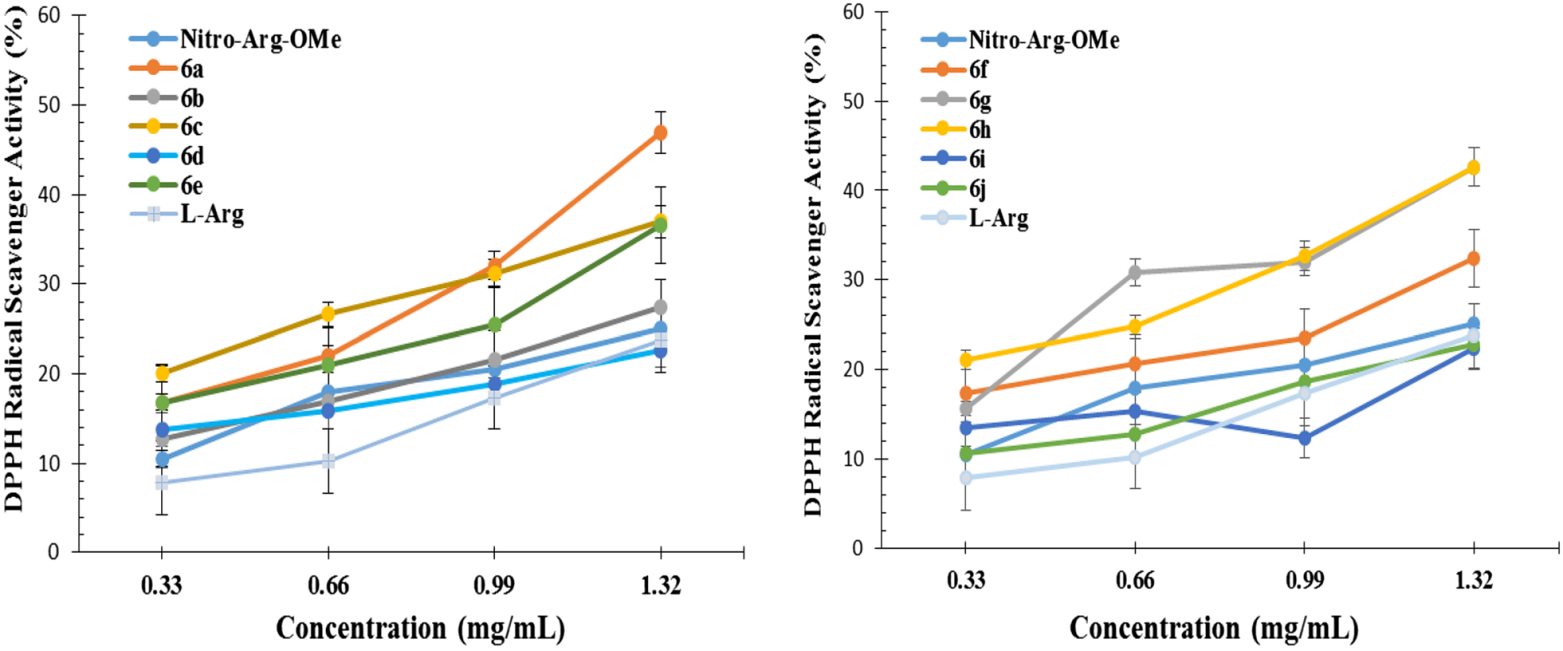
The DPPH radical scavenging ability (%) of derivatives **6a**–**j**

The purple free radical DPPH (2,2-diphenyl-1-(2,4,6-trinitrophenyl)hydrazyl) is a stable compound that can be scavenged through antioxidants by reduction to 2,2-diphenyl-1-(2,4,6-trinitrophenyl)hydrazine), a colorless or yellow product visible at 517 nm [32]. The scavenging activities (%) of thiazolidine-4-one derivatives of nitro-l-arginine methyl ester **6a**–**j** at different concentrations (0.33, 0.66, 0.99 and 1.32 mg/mL) are presented in Fig. 1. The high values of the scavenging activity indicate a good antiradical effect. The results expressed as EC_50_ values (mg/mL) are shown in Table 2. Low values of EC_50_ demonstrate a higher scavenging ability.

**Table 2:** The DPPH scavenging ability (EC_50_ mg/mL) of derivatives **6a**–**j**

Compound	EC_50_ (mg/mL)	Compound	EC_50_ (mg/mL)
**6a**	1.7294 ± 0.048	**6g**	1.8869 ± 0.013
**6b**	2.5980 ± 0.013	**6h**	1.8068 ± 0.028
**6c**	2.5354 ± 0.021	**6i**	2.7992 ± 0.012
**6d**	2.6176 ± 0.012	**6j**	2.8034 ± 0.014
**6e**	2.2430 ± 0.032	**NO**_**2**_ **-Arg-OMe**	2.7163 ± 0.019
**6f**	2.4751 ± 0.015	**L-Arg**	2.8157 ± 0.017
**Vitamin E**	0.0018 ± 0.008		

Data are mean ± SD (n = 3, p < 0.05)

It was observed that 1,3-thiazolidine-4-one derivatives of methyl ester of nitro-l-arginine (NO_2_-Arg-OMe) showed an improved scavenging ability compared to parent molecule (NO_2_-Arg-OMe) and l-arginine, excepting nitro substituted derivatives **6i** and **6j**, which showed comparable antiradical activity. It is also noted that the antiradical activity increases with the concentration, the highest inhibition being recorded at the concentration of 1.32 mg/mL. At this concentration the inhibition rate ranged from 22.62 % for **6d** (R = 4-F) up to 42.61 % for **6h** (R = 2-OCH_3_) and 47.63 % for **6a** (R = H).

The scavenging ability depends on the substituent of phenyl ring of thiazolidine-4-one moiety. The most active compound was unsubstituted derivative **6a** (EC_50_ = 1.7294 ± 0.048), which is 1.6 times more active than NO_2_-Arg-OMe (EC_50_ = 2.7163 ± 0.019). A good influence was showed also by the methoxy substitution in *ortho* and *meta* position, the corresponding compounds **6h** (2-OCH_3_, EC_50_ = 1.8068 ± 0.028) and **6g** (3-OCH_3_, EC_50_ = 1.8868 ± 0.013) being 1.5 times more active than NO_2_-Arg-OMe. All tested compounds were less active than vitamin E used as a positive control.

#### The ABTS radical scavenging assay

The radical of 2,2′-azinobis-(3-ethylbenzothiazoline-6-sulfonic acid) (ABTS^·+^) generated by oxidation of ABTS with potassium persulfate is reduced in the presence of hydrogen-donating compounds. The influence of concentration of the antioxidant and duration of reaction on the radical cation absorption inhibition are taken into account for antioxidant activity evaluation [33]. The antioxidants produce a discoloration with a decrease in the absorbance measured at 734 nm [34].

**Fig. 2:**
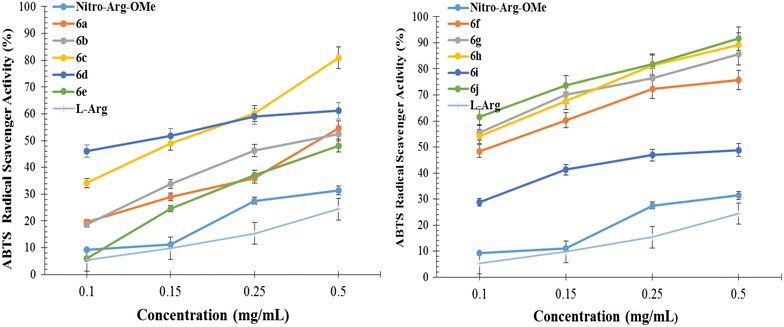
The ABTS radical scavenging ability (%) of derivatives **6a**–**j**

The ABTS radical scavenging ability (%) of **6a**–**j** at different concentrations (0.1, 0.15, 0.25, 0.5 mg/mL) are presented in Fig. 2. The high values of scavenging activity indicate a good antiradical effect. The results expressed as EC_50_ values (mg/mL) are presented in Table 3. Low values of EC_50_ indicate a higher effectiveness in ABTS scavenging ability.

**Table 3:** The ABTS scavenging ability (EC_50_ mg/mL) of derivatives **6a**–**j**

Compound	EC_50_ (mg/mL)	Compound	EC_50_ (mg/mL)
**6a**	0.4699 ± 0.013	**6g**	0.0827 ± 0.017
**6b**	0.4967 ± 0.015	**6h**	0.0918 ± 0.032
**6c**	0.1885 ± 0.014	**6i**	0.9434 ± 0.018
**6d**	0.1720 ± 0.018	**6j**	0.0525 ± 0.015
**6e**	0.5954 ± 0.029	**NO**_**2**_ **-Arg-OMe**	1.8487 ± 0.026
**6f**	0.4182 ± 0.012	**L-Arg**	2.0574 ± 0.011
**Vitamin E**	0.0075 ± 0.008		

Data are mean ± SD (n = 3, p < 0.05)

The data showed that ABTS^·+^ is inhibited in a higher rate than DPPH radical, all derivatives being more active than parent compound. This means that the chemical modulation made on the NO_2_-Arg-OMe scaffold improves the radical scavenging activity. The radical scavenging ability increases with the concentration, the highest inhibition being recorded at the concentration of 0.5 mg/mL (Fig. 2). At this concentration the inhibition rate ranged from 48.15 % for **6e** (R = 4-Br) up to 89.26 % for **6h** (R = 3-NO_2_) and 91.55 % for **6j** (R = 2-NO_2_), the inhibition percentage being approximately 2 times higher than the DPPH inhibition percentage.

The activity is depending on the substitution of phenyl ring of thiazolidine-4-one scaffold (Table 3). The most active compounds were **6j**, **6g** and **6h** that have nitro in *ortho* position and methoxy in *ortho* and *para* position respectively. These compounds are 35 times (**6j**, EC_50_ = 0.0525 ± 0.015), 22 times (**6g**, EC_50_ = 0.0827 ± 0.017) and 20 times (**6h**, EC_50_ = 0.0918 ± 0.032) more active than NO_2_-Arg-OMe (EC_50_ = 1.8487 ± 0.026). A very good activity was showed also by the compounds **6c** and **6d** that have chloro and fluoro in *para* postion of phenyl ring. They are 10 times (**6c**, EC_50_ = 0.1885 ± 0.014) and 11 times (**6d**, EC_50_ = 0.1720 ± 0.018) respectively more active than NO_2_-Arg-OMe. It is also noted that all tested compounds are more active than l-arginine but less active than vitamin E used as a positive control.

#### Phosphomolydenum reducing antioxidant power (PRAP) assay

**Fig. 3:**
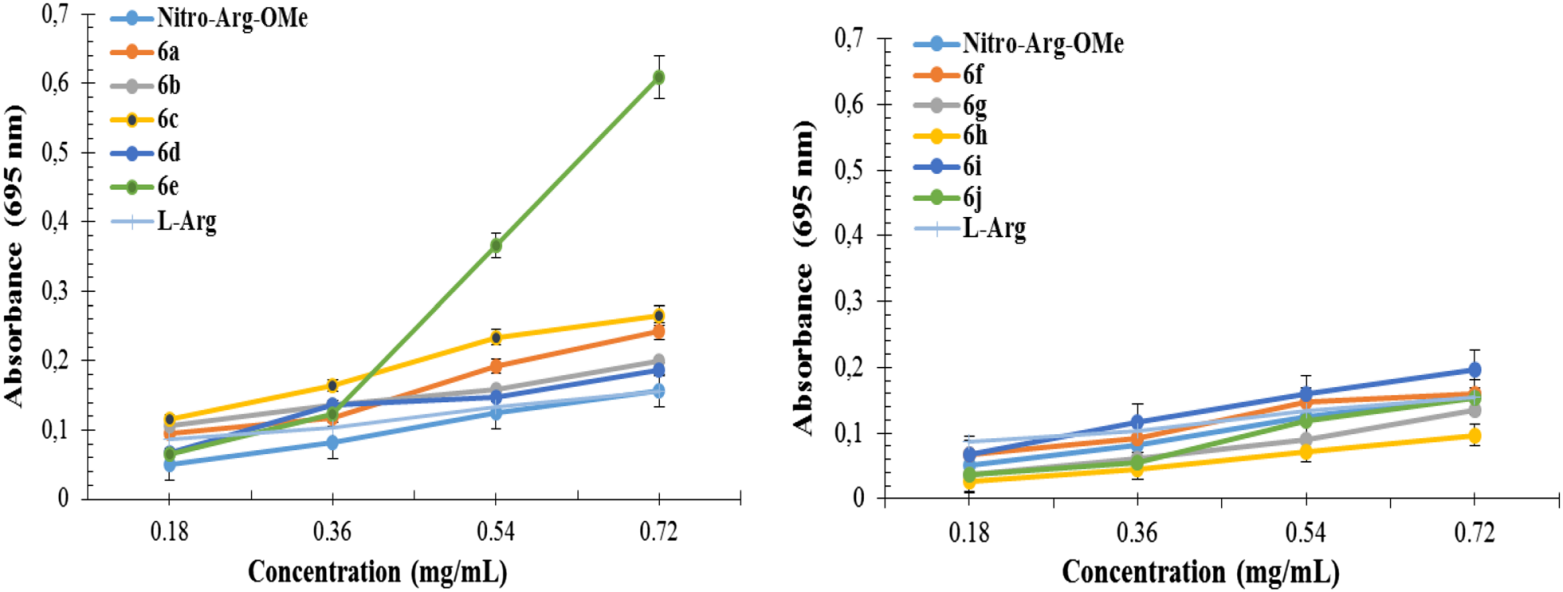
The absorbance of derivatives **6a**–**j** in reference with NO_2_-Arg-OMe

The total antioxidant activity was determined by the formation of phosphomolybdenum blue complex by the reduction of Mo^6+^ to Mo^5+^ under the action of electron donating compounds. The maximum absorption of the complex was recorded at 695 nm and the reducing antioxidant effectiveness is correlated with high absorbance values [35]. The graphical representation of the absorbance values at different concentrations (0.18, 0.36, 0.54 and 0.72 mg/mL) is shown in Fig. 3. As we expected, the absorbance of **6a**–**j** increases with the concentration, the highest absorbance/activity being recorded at the concentration of 0.72 mg/mL.

The data support the positive influence of thiazolidine-4-one moiety for increase the antioxidant effect of NO_2_-Arg-OMe, the corresponding compound **6a** (EC_50_ = 1.6235 ± 0.015) being 1.6 times more active than NO_2_-Arg-OMe (EC_50_ = 2.6169 ± 0.032) (Table 4). Regarding the influence of radicals which substitute the phenyl ring from thiazolidine-4-one it was observed that the most favorable influence was exerted by the substitution in *para* with Br, the corresponding compound **6e,** (EC_50_ = 0.6405 ± 0.012) being 4 times more active than the NO_2_-Arg-OMe. Although the activity of the all tested compounds is more intense than l-arginine, they are less active than vitamin E used as a positive control.

**Table 4:** The phosphomolydenum reducing antioxidant power (EC_50_ mg/mL) of **6a**–**j** derivatives

Compound	EC_50_ (mg/mL)	Compound	EC_50_ (mg/mL)
**6a**	1.6235 ± 0.015	**6g**	2.7332 ± 0.037
**6b**	2.0679 ± 0.018	**6h**	3.5186 ± 0.018
**6c**	2.0734 ± 0.022	**6i**	2.1837 ± 0.024
**6d**	2.1706 ± 0.014	**6j**	2.4610 ± 0.019
**6e**	0.6405 ± 0.012	**NO**_**2**_ **-Arg-OMe**	2.6169 ± 0.032
**6f**	2.3827 ± 0.013	**L-Arg**	2.7534 ± 0.006
**Vitamin E**	0.0385 ± 0.001		

Data are mean ± SD (n = 3, p < 0.05)

#### Ferric reducing antioxidant power (FRAP) assay

**Fig. 4:**
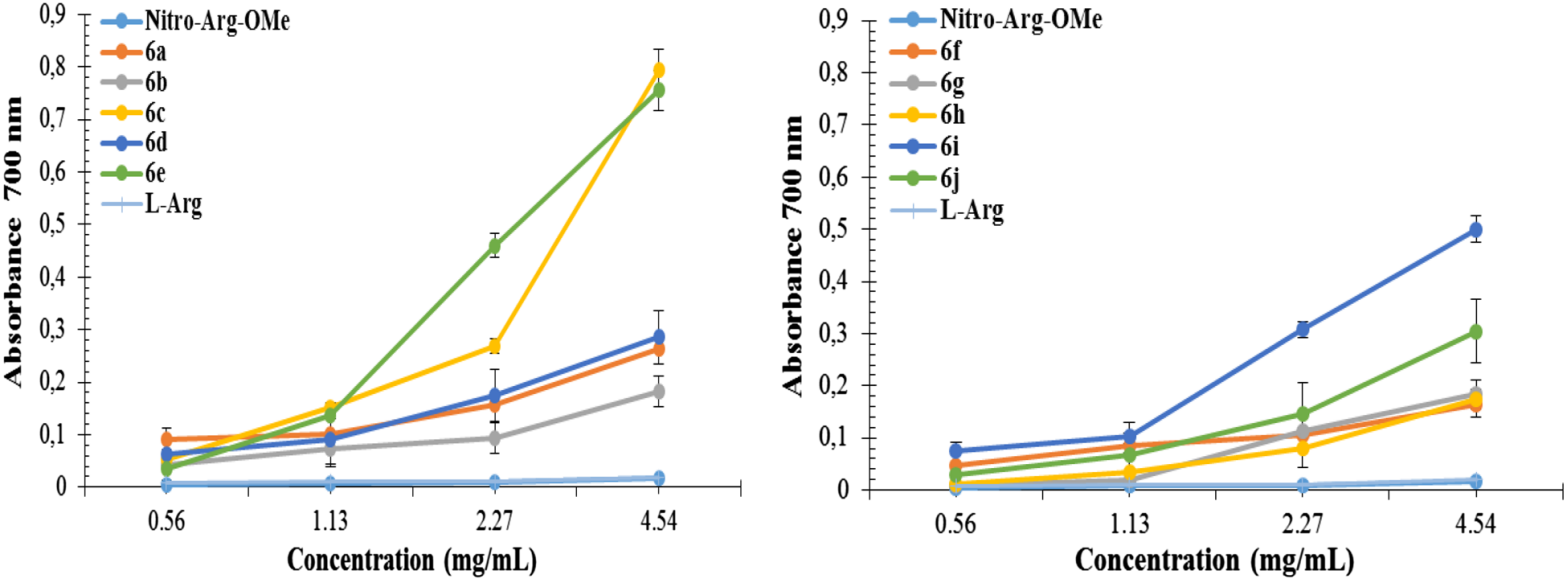
The absorbance of derivatives **6a**–**j** in reference with NO_2_-Arg-OMe

The ferric reducing antioxidant power assay is a sensitive method based on the reduction of ferricyanide to ferrocyanide in the presence of antioxidants with electron-donating abilities. Ferrocyanide is quantified as Perl’s Prussian Blue, complex which has a maximum absorption band at 700 nm [36]. The absorbance values of our compounds at different concentrations (0.56, 1.13, 2.27, 4.54 mg/mL) are shown in Fig. 4 and the EC_50_ values are presented in Table 5.

**Table 5:** The ferric reducing antioxidant power (EC_50_, mg/mL) of **6a**–**j**

Compound	EC_50_ (mg/mL)	Compound	EC_50_ (mg/mL)
**6a**	7.1876 ± 0.038	**6g**	4.6474 ± 0.018
**6b**	9.0695 ± 0.015	**6h**	7.9317 ± 0.023
**6c**	3.2742 ± 0.019	**6i**	4.5202 ± 0.014
**6d**	8.9671 ± 0.023	**6j**	7.3504 ± 0.011
**6e**	2.5781 ± 0.012	**NO**_**2**_ **-Arg-OMe**	11.0778 ± 0.016
**6f**	6.1302 ± 0.032	**L-Arg**	10.9321 ± 0.015
**Vitamin E**	0.0109 ± 0.003		

Data are mean ± SD (n = 3, p < 0.05)

The derivatization of NO_2_-Arg-OMe through an introduction of thiazolidine-4-one moiety via amide chain has a great influence on antioxidant potential, all the tested compounds being more active than parent molecule (NO_2_-Arg-OMe) and l-arginine. The most active compounds were **6e** (EC_50_ = 2.5781 ± 0.012) and **6c** (EC_50_ = 3.2742 ± 0.019) which contain bromo and chloro in *para* position of phenyl ring. These compounds were 4.5 times and 3.4 times respectively more active than NO_2_-Arg-OMe (EC_50_ = 11.0778 ± 0.016). A good influence was produced also by substitution in *meta* position with methoxy and nitro, the corresponding compounds being 2.5 times (**6i,** EC_50_ = 4.5202 ± 0.014) and 2.4 times (**6g**, EC_50_ = 4.6474 ± 0.018) more active than NO_2_-Arg-OMe. All tested compounds were less active than vitamin E used as a positive control.

#### Antibacterial/antifungal assays

The antibacterial and antifungal activity of our derivatives was evaluated using the agar disc diffusion method and broth micro-dilution method.

#### The agar disc diffusion method

The data presented in Table 6 show that tested compounds are active on both bacterial and fungal strains, their effect being more intense or comparable with parent molecule (NO_2_-Arg-OMe). The main characteristic of the tested compounds is their activity on *P. aeruginosa* ATCC 27853, a Gram-negative bacterial strain frequently found in wounds. This effect is important because Gram-negative bacteria are more resistant than Gram-positive ones to the treatment due to lipopolysaccharide-rich outer membrane which significantly reduces the intracellular penetration of antibiotics [36, 37]. It is noted that in similar experimental conditions, ampicillin and chloramphenicol, used as standard drugs, were inactive on *P. aeruginosa* ATCC 27853, the data being in agreement with other experimental studies [38, 39]. The most proper compound seems to be **6j** which has nitro in *ortho* position of phenyl ring. This compound was the most active against *S. aureus*, *Sarcina lutea* and *P. aeruginosa* strains in comparation with NO_2_-Arg-OMe (**5**).

**Table 6:** Antibacterial/antifungal inhibition area (mm) of **6a**–**j** derivatives

Sample	Diameter of inhibition area^a^ (mm)						
	*Bacterial strains*				*Yeasts strains*		
	SA	SL	EC	PA	CA	CG	CP
**6a**	15.2 ± 0.12	19.3 ± 0.15	10.1 ± 0.06	13.1 ± 0.24	11.8 ± 0.35	15.2 ± 0.28	23.0 ± 0.19
**6b**	14.1 ± 0.08	20.1 ± 0.13	15.1 ± 0.23	11.2 ± 0.41	12.9 ± 0.06	15.2 ± 0.98	24.1 ± 0.65
**6c**	15.2 ± 0.16	18.1 ± 0.78	11.2 ± 0.63	11.9 ± 0.09	9.9 ± 0.62	13.8 ± 0.07	21.2 ± 0.33
**6d**	15.3 ± 0.68	18.2 ± 0.55	10.2 ± 0.37	–	13.2 ± 0.21	16.4 ± 0.78	24.2 ± 0.35
**6e**	12.1 ± 0.09	20.1 ± 0.43	10.1 ± 0.32	11.1 ± 0.19	12.1 ± 0.58	15.9 ± 0.55	25.3 ± 0.28
**6f**	15.2 ± 0.52	20.1 ± 0.26	12.2 ± 1.05	10.2 ± 0.36	12.1 ± 0.18	15.5 ± 0.48	25.1 ± 0.37
**6g**	13.1 ± 0.15	20.1 ± 0.72	–	10.1 ± 0.09	12.1 ± 0.28	15.9 ± 1.07	25.2 ± 0.39
**6h**	14.1 ± 0.09	20.3 ± 0.43	11.1 ± 0.30	10.2 ± 0.15	12.1 ± 0.86	13.8 ± 0.57	23.1 ± 0.22
**6i**	12.3 ± 0.08	21.1 ± 0.13	10.1 ± 0.23	12.2 ± 0.41	15.4 ± 0.06	16.4 ± 0.98	20.1 ± 0.65
**6j**	16.3 ± 0.34	21.2 ± 0.87	10.2 ± 0.51	13.1 ± 0.82	15.2 ± 0.74	15.2 ± 0.32	23.1 ± 0.47
**5**	14.9 ± 0.16	19.9 ± 0.12	11.9 ± 0.06	11.8 ± 0.19	13.8 ± 0.15	15.9 ± 0.17	19.9 ± 0.09
**A**	20.1 ± 0.57	21.2 ± 1.16	15.2 ± 0.67	–	–	–	–
**C**	16.3 ± 0.28	30.4 ± 0.35	20.1 ± 0.16	–	–	–	–
**N**	–	–	–	–	19.4 ± 0.51	19.5 ± 0.72	12.4 ± 0.42

SA = *Staphylococcus aureus* ATCC 25923; SL = *Sarcina lutea* ATCC 9341; EC = *Escherichia coli* ATCC 25922; PA = *Pseudomonas aeruginosa* ATCC 27853; CA = *Candida albicans* ATCC 10231; CG = *Candida glabrata* ATCC MYA 2950; CP = *Candida parapsilosis* ATCC 22019; 5 = NO_2_-Arg-OMe; A = ampicillin; C = chloramphenicol; N = nystatin. **5** = L-NO_2_-Arg-OMe

^a^Mean values (n = 3) ± standard deviation

Regarding the antifungal activity the data support the positive influence of *nitro* substitution of phenyl ring, the corresponding compounds being more active than NO_2_-Arg-OMe, especially on *Candida albicans* (**6i,** R = 3-NO_2_**, 6j,** R = 2-NO_2_) and *Candida glabrata****(*6i, R** **=** 3-NO_2_*).* On *C. glabrata* a good activity was showed also by **6d** (R = 4-F). Referring to *Candida parapsilosis* strain it is noted that all tested compounds were more active than parent compound (NO_2_-Arg-OMe, **5**) and nystatin.

#### The broth micro-dilution method

After the antimicrobial activity was proved, the next step was to establish the minimal inhibitory concentration (MIC) and the minimal bactericidal/fungicidal concentration (MBC/MFC) using the broth micro-dilution method.

The antibacterial activity of **6j** is supported by the MIC and MBC values (Table 7); this compound having smaller values than NO_2_-Arg-OMe for *S. aureus* and *Escherichia coli*. A good activity against these bacterial strains was also showed by the **6c**, which contains chloro in *para* position of phenyl ring of thiazolidine-4-one moiety. The data support also the antibacterial effect of **6i** and **6f** against *P. aeruginosa*, their MIC and MBC values being smaller than NO_2_-Arg-OMe.

**Table 7:** Antibacterial effect expressed as MIC and MBC values (mg/mL) of **6a**–**j**

Sample	*S. aureus* ATCC 25923		*S. lutea* ATCC 9341		*E. coli* ATCC 25922		*P. aeruginosa* ATCC 27853	
	MIC^a^	MBC^a^	MIC^a^	MBC^a^	MIC^a^	MBC^a^	MIC^a^	MBC^a^
**6a**	2.5	2.5	0.01	0.01	1.25	1.25	2.5	2.5
**6b**	1.25	2.5	0.03	1.25	2.5	10	2,5	10
**6c**	0.01	0.01	0.01	0.03	0.03	0.03	5	5
**6d**	2.5	2.5	0.01	0.03	1.25	5	1.25	1.25
**6e**	0.01	0.3	0.01	0.01	10	5	5	5
**6f**	0.07	2.5	0.03	0.15	0.03	0.15	0.03	1.25
**6g**	2.5	2.5	0.03	1.25	1.25	1.25	2.5	10
**6h**	2.5	2.5	0.01	0.01	1.25	5	2.5	2.5
**6i**	0.3	0.3	0.01	0.01	0.1	0.1	0.03	0.6
**6j**	0.07	0.07	0.01	0.01	0.03	0.03	1.25	1.25
**5**	2.5	2.5	0.01	0.01	1.25	1.25	1.25	1.25
**A**	0.0002	0.0005	0.0002	0.0005	0.008	0.016	nt	nt
**C**	0.008	0.016	0.003	0.006	0.008	0.016	nt	nt

**5** = L-NO_2_-Arg-OMe, A = ampicillin; C = chloramphenicol; nt = no tested

^a^Mean values (n = 3) ± standard deviation

Although the results obtained using agar disc diffusion method support that some of tested compounds are more active than positive control (ampicillin and chloramphenicol), this observation has not been proved by the MIC and MBC values. All tested compounds were less active ampicillin and chloramphenicol on tested bacterial strains, except *P. aeruginosa* ATCC 27853.

The results obtained for antifungal activity (Table 8) support the selectivity of the almost tested compounds, included the parent compound (NO_2_-Arg-OMe), on *C. parapsilosis* strain. For this strain the MIC values of almost tested compounds were comparable with nystatin while the MFC values were even lower than it. The data support also the activity of **6i** on *C. albicans* in comparation with NO_2_-Arg-OMe.

**Table 8:** Antifungal effect expressed as MIC and MFC values (mg/mL) of **6a**–**j**

Sample	*C. albicans* ATCC 10231		*C. glabrata* ATCC MYA 2950		*C. parapsilosis* ATCC 22019	
	MIC^a^	MFC^a^	MIC^a^	MFC^a^	MIC^a^	MFC^a^
**6a**	0.6	1.25	1.25	1.25	0.003	0.003
**6b**	0.6	1.25	2.5	10	0.003	0.003
**6c**	0.6	0.6	10	10	0.003	0.003
**6d**	0.3	1.25	0.6	2.5	0.003	0.003
**6e**	10	10	10	10	5	5
**6f**	0.6	0.6	2.5	5	0.003	0.003
**6g**	0.3	1.25	1.25	2.5	0.003	0.003
**6h**	0.3	5	10	10	0.003	0.003
**6i**	0.03	10	10	10	10	10
**6j**	0.3	1.25	1.25	1.25	0.003	0.003
**5**	1.25	1.25	0.6	2.5	0.003	0.003
**N**	0.004	0.008	0.004	0.008	0.004	0.008

**5** = -L-NO_2_-Arg-OMe, N = nystatin

^a^Mean values (n = 3) ± standard deviation

### Experimental section

#### General methods

All chemicals used for the synthesis of the desired compounds were obtained from Sigma Aldrich Company and Fluka Company and were used as received without additional purification. The melting points were measured using a Buchi Melting Point B-540 apparatus and they are uncorrected. The FT-IR spectra were recorded on Horizon MBTM FT-IR, over a 500–4000 cm^−1^ range, after 16 scans at a resolution of 4 cm^−1^. The spectra processing was carried out with the Horizon MBTM FTIR Software. The ^1^H-NMR (400 MHz) and ^13^C-NMR (101 MHz) spectra were obtained on a Bruker Avance 400 MHz spectrometer using tetramethylsilane as internal standard and deuterated chloroform as solvent (CDCl_3_). The chemical shifts were shown in δ values (ppm). The mass spectra were registered using a Bruker MaXis Ultra-High Resolution Quadrupole Time-of-Flight Mass Spectrometer. The progress of the reaction was monitored on TLC, using pre-coated Kieselgel 60 F254 plates (Merck, Whitehouse Station, NJ, USA) and the compounds were visualized using UV light. E-factor and material efficiency (ME) have been selected to evaluate the greenness of the synthetic procedures. E-factor is a very useful metric tool that is defined as E-Factor = mass of wastes/mass of product. The E-factor can be used to calculate the material efficiency of the process according to the equation: ME = 1/E-factor + 1 [40].

The antioxidant potential was investigated using in vitro methods based on ferric/phosphomolybdenum reducing antioxidant power and DPPH/ABTS radical scavenging assay. The antibacterial activity was evaluated using Gram-positive (*S. aureus* ATCC 25923, *S. lutea* ATCC 9341) and Gram-negative (*E. coli* ATCC 25922 and *P. aeruginosa* ATCC 27853) bacterial strains. The antifungal activity was evaluated using *C. albicans* ATCC 10231, *C. glabrata* ATCC MYA 2950 and *C. parapsilosis* ATCC 22019. All strains were obtained from the Culture Collection of the Department of Microbiology, Gr. T. Popa University of Medicine and Pharmacy, Iasi, Romania. As positive controls were used ampicillin, a beta-lactam drug, and chloramphenicol which belongs amphenicoles class for antibacterial activity and nystatin for antifungal activity.

### General procedure for synthesis of *N*^2^-[(2-aryl-4-oxo-1,3-thiazolidin-3-yl)propionyl]-nitro-l-arginine methyl ester (**6a**–**j**)

3-(2-Phenyl-4-oxo-1,3-thiazolidin-3-yl)propionic acid derivatives, **4a**–**j** (5 mmol) were dissolved in 25 mL freshly distilled DCM, on ice bath at 0–5 °C and under inert atmosphere of nitrogen [41]. To the cold solution it was added EDCI.HCl (5.5 mmol, 1.1 equiv.), HOBt (5.5 mmol, 1.1 equiv.) and NO_2_-L-Arg-OMe.HCl (5.5 mmol, 1.1 equiv.). The mixture was stirred for 10–14 h at room temperature. The reaction monitoring was carried out by Thin Layer Chromatography (TLC) using as mobile phase DCM: methanol (MeOH) = 9.5: 0.5 (v/v) and the spot visualization was done under UV light at 254 nm. After the completion of the reaction, the mixture was washed successively with 1 M HCl, saturated solution of sodium bicarbonate and saturated brine solution. The organic layer, was dried over anhydrous MgSO_4_, filtered and concentrated to dryness. Purification of compounds was carried out by column separation on silica gel (DCM/MeOH, 9.5/0.5). The appropriate fractions of thiazolidine-4-one derivatives was collected and then evaporated to dryness to give the corresponding final derivatives.

### *N*^2^-[(2-Phenyl-4-oxo-1,3-thiazolidin-3-yl)propionyl]-nitro-l-arginine methyl ester (6a)

White cristals, mp 102 °C, yield: 93 %, IR (Zn/Se crystal, cm^−1^): 3294 (–NH); 2963, 783 (=CH_phenyl_); 2869, 1250, 725 (–CH_2_–); 1736 (COOCH_3_); 1647 (CONH); 1628 (C=O_thiazolidine-4-one_); 1535 (–C=C–_phenyl_); 1350, 1026 (–C–N–); 698 (C–S); ^1^H-NMR (δ ppm): 8.51 (s, 1H, NH–CO), 8.03 (m, 1H, NH), 7.56–7.47 (m, 2H, NH), 7.38–7.29 (m, 5H, Ar–H), 5.77 (d, J = 55.7 Hz, 1H, –N–CH–S), 4.61 (s, 1H, CH_2_–S), 3.89 (s, 1H, CH_2_–S), 3.78 (s, 3H, CH_3_ ester), 3.73 (s, 1H, CH–COOCH_3_), 3.69 (s, 1H, N–CH_2_), 3.39–3.30 (m, 2H, CH_2_ arg), 3.23–3.01 (m, 1H, N–CH_2_), 2.62–2.34 (m, 2H, CH_2_–CO), 1.94–1.54 (m, 4H, 2CH_2_ arg); ^13^C-NMR (δ ppm): 172.32, 171.28, 162.09 (3C, CO), 159.64 (C_guanid_), 139.15, 129.72, 129.42, 127.45, 127.33, 117.60 (6C, C_Ar_), 64.36 (S–CH–N–), 52.99 (CH_2_), 48.47 (CH), 39.75 (–CH_2_N–), 33.99 (–CH_2_S–), 33.33 (CH_2_), 32.98 (–CH_2_CO), 24.29 (CH_2_), 20.57 (CH_3_); HRMS (EI-MS): *m*/*z* calculated for C_19_H_26_N_6_O_6_S [M + H]^+^ 467.1707; found is 467.1705; Green chemistry metrics: E-factor 22.513, ME 0.042.

### *N*^2^-[(2-(4-Methylphenyl)-4-oxo-1,3-thiazolidin-3-yl)propionyl]-nitro-l-arginine methyl ester (**6b**)

Light yellow cristals, mp 90 °C, yield: 91 %, IR (Zn/Se crystal, cm^−1^): 3305 (–NH); 2951, 771 (=C–H_phenyl_); 2928, 1257, 721 (–CH_2_–); 1724 (COOCH_3_); 1678 (CONH); 1628 (C=O_thiazolidine-4-one_); 1597 (–C=C–_phenyl_); 1362, 1026 (–C–N–); 694 (C–S); ^1^H-NMR (δ ppm): 8.68 (s, 1H, NH–CO), 8.31 (m, 1H, NH), 7.80 (s, 2H, NH), 7.26–7.34 (m, 4H, Ar–H), 5.82 (d, J = 18.8 Hz, 1H, –N–CH–S), 4.57 (s, 1H, CH_2_–S), 3.92 (dd, J = 13.6, 6.8 Hz, 1H, CH_2_–S), 3.81 (s, 3H, CH_3_ ester), 3.78 (s, 1H, CH–COOCH_3_), 3.71 (s, 1H, N–CH_2_), 3.53–3.31 (m, 2H, CH_2_ arg), 3.24–3.05 (m, 1H, N–CH_2_), 2.62 (dd, J = 18.0, 7.9 Hz, 2H, CH_2_–CO), 2.41 (s, 3H, CH_3_), 2.02–1.62 (m, 4H, 2CH_2_ arg); ^13^C-NMR (δ ppm): 172.47, 171.28, 170.76 (3C, CO), 159.40 (C_guanid_), 138.24, 134.57 (2C, C_Ar_), 128.95 (2C, CH_Ar_), 123.31 (2C, CH_Ar_), 63.25 (S–CH–N), 50.34 (CH), 40.73 (CH_2_), 39.58 (–CH_2_N–), 33.45 (–CH_2_S–), 32.84 (–CH_2_CO), 29.14 (CH_2_), 24.29 (CH_2_), 26.37, 21.34 (2C, CH_3_); HRMS (EI-MS): *m*/*z* calculated for C_20_H_28_N_6_O_6_S [M + H]^+^ 481.1862; found 481.1864; Green chemistry metrics: E-factor 16.891, ME 0.056.

### *N*^2^-[(2-(4-Chlorophenyl)-4-oxo-1,3-thiazolidin-3-yl)propionyl]-nitro-l-arginine methyl ester (**6c**)

Light yellow cristals, mp 146 °C, yield: 89 %; IR (Zn/Se crystal, cm^−1^): 3302 (–NH); 2951, 783 (=C–H_phenyl_); 2928, 1257, 725 (–CH_2_–); 1736 (COOCH_3_); 1651 (–CONH); 1628 (C=O_thiazolidine-4-one_); 1597 (–C=C–_phenyl_); 1342, 1014 (–C–N–);764 (C–Cl); 683 (C–S); ^1^H-NMR (δ ppm): 8.68 (s, 1H, NH–CO), 8.26 (m, 1H, NH), 7.75 (s, 2H, NH), 7.32 (d, J = 8.2 Hz, 2H, Ar–H), 7.28–7.23 (d, 2H, Ar–H), 5.75 (d, J = 26.3 Hz, 1H, –N–CH-S), 4.52 (s, 1H, CH_2_–S), 3.79 (dd, J = 15.8, 8.6 Hz, 1H, CH–COOCH_3_), 3.71 (s, 1H, CH_2_–S), 3.68 (s, 3H, CH_3_ ester), 3.64 (s, 1H, N–CH_2_), 3.31 (d, J = 44.9 Hz, 2H, CH_2_ arg), 3.11–2.94 (m, 1H, N–CH_2_), 2.65–2.29 (m, 2H, CH_2_–CO), 1.90–1.56 (m, 4H, 2CH_2_ arg); ^13^C-NMR (δ ppm): 173.24, 171.99, 169.52 (3C, CO), 160.48 (C_guanid_), 138.42, 135.80 (C_Ar_), 130.05 (2C, CH_Ar_), 129.37 (2C, CH_Ar_), 63.94 (S–CH–N), 53.38 (CH_2_), 51.34 (CH), 41.42 (CH_2_), 39.10 (–CH_2_N–), 34.19 (–CH_2_S–), 31.53 (–CH_2_CO), 29.57 (CH_2_); 26.45 (CH_3_); HRMS (EI-MS): *m*/*z* calculated for C_19_H_25_ClN_6_O_6_S [M + H]^+^ 501.1317; found 501.1310; Green chemistry metrics: E-factor 2.361, ME 0.297.

### *N*^2^-[(2-(4-Fluorophenyl)-4-oxo-1,3-thiazolidin-3-yl)propionyl]-nitro-l-arginine methyl ester (**6d**)

Light yellow cristals, mp 85 °C, yield: 75 %; IR (Zn/Se crystal, cm^−1^): 3302 (–NH); 2951, 787 (=C–H_phenyl_); 2933, 1257, 725 (–CH_2_–); 1736 (COOCH_3_); 1651 (–CONH); 1647 (C=O_thiazolidine-4-one_); 1601 (–C=C–_phenyl_); 1342, 1011 (–C–N–); 1153 (C–F); 687 (C–S); ^1^H-NMR (δ ppm): 8.63 (s, 1H, NH–CO), 8.24 (m, 1H, NH), 7.61 (s, 2H, NH), 7.37 (dd, J = 13.7, 5.7 Hz, 2H, Ar–H), 7.11 (t, J = 8.4 Hz, 2H, Ar–H), 5.80 (d, J = 50.0 Hz, 1H, –N–CH–S), 4.72–4.41 (m, 1H, CH_2_–S), 3.94–3.85 (m, 1H, CH–COOCH_3_), 3.80 (s, 1H, CH_2_–S), 3.75 (s, 3H, CH_3_ ester), 3.71 (s, 1H, N–CH_2_), 3.52–3.27 (m, 2H, CH_2_ arg), 3.20–3.00 (m, 1H, N–CH_2_), 2.68–2.27 (m, 2H, CH_2_–CO), 1.84–1.59 (m, 4H, 2CH_2_ arg); ^13^C-NMR (δ ppm): 171.80, 170.76, 162.34 (3C, CO), 158.81 (C_guanid_), 161.58, 135.57 (2C, C_Ar_), 128.32 (2C, CH_Ar_), 115.61 (2C, CH_Ar_), 62.65 (S–CH–N), 52.15 (CH), 39.93 (–CH_2_N–), 38.85 (CH_2_), 33.10 (–CH_2_S–), 32.16 (–CH_2_CO), 29.24 (CH_2_), 28.67 (CH_2_), 21.34 (CH_3_); HRMS (EI-MS): *m*/*z* calculated for C_19_H_25_FN_6_O_6_S [M + H]^+^ 485.1614; found 485.1613; Green chemistry metrics: E-factor 1.122, ME 0.471.

### *N*^2^-[(2-(4-Bromophenyl)-4-oxo-1,3-thiazolidin-3-yl)propionyl]-nitro-l-arginine methyl ester (**6e**)

Light yellow cristals, mp 109 °C, yield: 87 %; IR (Zn/Se crystal, cm^−1^): 3294 (–NH); 2954, 776 (=C–H_phenyl_); 1736 (COOCH_3_); 1647 (–CONH); 1628 (C=O_thiazolidine-4-one_); 1601 (–C=C–_phenyl_); 1342, 1007 (–C–N–); 1246, 725 (–CH_2_–); 687 (C–S); 668 (C–Br); ^1^H-NMR (δ ppm): 8.68 (s, 1H, NH–CO), 8.19 (m, 1H, NH), 7.76 (s, 2H, NH), 7.54 (d, J = 7.6 Hz, 2H, Ar–H), 7.36–7.16 (m, 2H, Ar–H), 5.79 (d, J = 29.5 Hz, 1H, –N–CH–S), 4.59 (s, 1H, CH_2_–S), 3.86 (dd, J = 17.6, 10.2 Hz, 1H, CH_2_–S), 3.78 (s, 3H, CH_3_ ester), 3.77 (s, 1H, CH–COOCH_3_), 3.72 (d, J = 15.6 Hz, 1H, N–CH_2_), 3.37 (d, J = 45.7 Hz, 2H, CH_2_ arg), 3.09 (dd, J = 31.1, 10.2 Hz, 1H, N–CH_2_), 2.74–2.51 (m, 1H, CH_2_–CO), 2.43–2,37 (m, 1H, CH_2_–CO), 1.82 (d, J = 78.0 Hz, 4H, 2CH_2_ arg); ^13^C-NMR (δ ppm): 172.47, 170.18, 161.28 (3C, CO), 159.40 (C_guanid_), 138.24, 132.34 (2C, C_Ar_), 128.95 (2C, CH_Ar_), 123.31 (2C, CH_Ar_), 63.25 (S–CH–N), 52.71 (CH), 40.73 (CH_2_), 39.58 (–CH_2_N–), 33.45 (–CH_2_S–), 32.04 (–CH_2_CO), 29.14 (CH_2_), 28,67 (CH_2_), 25.44 (CH_3_); HRMS (EI-MS): *m*/*z* calculated for C_19_H_25_BrN_6_O_6_S [M + H]^+^ 545.0811; found 545.0812; Green chemistry metrics: E-factor 1.874, ME 0.352.

### *N*^2^-[(2-(4-Methoxyphenyl)-4-oxo-1,3-thiazolidin-3-yl)propionyl]-nitro-l-arginine methyl ester (**6f**)

Light yellow cristals, mp 95 °C, yield: 86 %; IR (Zn/Se crystal, cm^−1^): 3298 (–NH); 3001, 783 (=C–H_phenyl_); 1740 (COOCH_3_); 1651 (–CONH); 1628 (C=O_thiazolidine-4-one_); 1609 (–C=C–_phenyl_); 1346, 1111 (–C–N–); 1246, 725 (–CH_2_–); 1153 (–OCH_3_); 687 (C–S); ^1^H-NMR (δ ppm): 8.60 (s, 1H, NH–CO), 8.21 (m, 1H, NH), 7.63 (s, 2H, NH), 7.38–7.18 (m, 2H, Ar–H), 6.91 (d, J = 8.6 Hz, 2H, Ar–H), 5.73 (d, J = 42.3 Hz, 1H, –N–CH–S), 4.65–4.53 (m, 1H, CH_2_–S), 3.90–3.84 (m, 1H, CH_2_–S), 3.82 (s, 3H, CH_3_ ester), 3.76 (d, J = 3.3 Hz, 3H, OCH_3_), 3.71 (s, 1H, CH–COOCH_3_), 3.52–3.27 (m, 2H, CH_2_ arg), 3.22–3.01 (m, 1H, N–CH_2_), 2.61–2.48 (m, 1H, N–CH_2_), 2.42–2.27 (m, 1H, CH_2_–CO), 1.95–1.85 (m, 1H, CH_2_–CO), 1.77–1.54 (m, 4H, 2CH_2_ arg); ^13^C-NMR (δ ppm): 172.40, 171.99, 160.63 (3C, CO), 159.62 (C_guanid_), 130.67, 130.11 (2C, C_Ar_), 128.96 (2C, CH_Ar_), 114.70 (2C, CH_Ar_), 63.80 (S–CH–N), 55.71 (CH), 52.92 (OCH_3_), 40.46 (CH_2_), 39.56 (–CH_2_N–), 33.94 (–CH_2_S–), 32.92 (CH_2_), 29.51 (–CH_2_CO), 23.68 (CH_2_), 21.45 (CH_3_); HRMS (EI-MS): *m*/*z* calculated for C_20_H_28_N_6_O_7_S [M + H]^+^ 497.1813; found 497.1813; Green chemistry metrics: E-factor 1.506, ME 0.403.

### *N*^2^-[(2-(3-Methoxyphenyl)-4-oxo-1,3-thiazolidin-3-yl)propionyl]-nitro-l-arginine methyl ester (**6g**)

Light pink cristals, mp 103 °C, yield: 78 %; IR (Zn/Se crystal, cm^−1^): 3298 (–NH); 3001, 771 (=C–H _phenyl_); 2951, 1254, 725 (–CH_2_–); 1740 (COOCH_3_); 1651 (-CONH); 1647 (C=O_thiazolidine-4-one_); 1601 (–C=C–_phenyl_); 1338, 1041 (–C–N–); 1149 (–OCH_3_); 694 (C–S); ^1^H-NMR (δ ppm): 8.52 (s, 1H, NH–CO), 8.09 (m, 1H, NH), 7.52–7.47 (m, 2H, NH), 7.32 (t, J = 7.9 Hz, 1H, Ar–H), 6.97–6.85 (m, 2H, Ar–H), 6.74 (dd, J = 31.2, 7.7 Hz, 1H, Ar–H), 5.72 (d, J = 63.5, 5.7 Hz, 1H, –N–CH–S), 4.68–4.54 (m, 1H, CH_2_–S), 3.94–3.85 (m, 1H, CH_2_–S), 3.82 (s, 3H, CH_3_ ester), 3.79–3.78 (d, J = 3.5 Hz, 3H, OCH_3_), 3.72 (s, 1H, CH–COOCH_3_), 3.57–3.29 (m, 2H, CH_2_ arg), 3.25–3.05 (m, 1H, N–CH_2_), 2.55 (dt, J = 7.6, 6.9 Hz, 1H, N–CH_2_), 2.45–2.32 (m, 1H, CH_2_–CO), 1.91 (dd, J = 8.5, 4.0 Hz, 1H, CH_2_–CO), 1.77–1.55 (m, 4H, 2CH_2_ arg); ^13^C-NMR (δ ppm): 172.37, 170.65, 160.39 (3C, CO), 159.66 (C_guanid_), 140.76, 130.52 (2C, C_Ar_), 119.47, 114.98, 114.75, 113.11 (4C, CH_Ar_), 64.07 (S–CH–N), 55.56 (CH), 53.01 (OCH_3_), 40.57 (CH_2_), 39.79 (–CH_2_N–), 34.05 (–CH_2_S–), 31.94 (–CH_2_CO), 29.65, 24.27 (2CH_2_), 21.34 (CH_3_); HRMS (EI-MS): *m*/*z* calculated for C_20_H_28_N_6_O_7_S [M + H]^+^ 497.1813; found 497.1812; Green chemistry metrics: E-factor 3.767, ME 0.213.

### *N*^2^-[(2-(2-Methoxyphenyl)-4-oxo-1,3-thiazolidin-3-yl)propionyl]-nitro-l-arginine methyl ester (**6h**)

Light yellow cristals, mp 115 °C, yield: 76 %; IR (Zn/Se crystal, cm^−1^): 3298 (–NH); 3078, 771 (=C–H _phenyl_); 2947, 1242, 725 (–CH_2_–); 1736 (COOCH_3_); 1647 (–CONH); 1628 (C=O_thiazolidine-4-one_); 1597 (–C=C–_phenyl_); 1350, 1049 (–C–N–); 1153 (–OCH_3_); 683 (C–S); ^1^H-NMR (δ ppm): 8.48 (s, 1H, NH–CO), 7.94 (m, 1H, NH), 7.50 (s, 2H, NH), 7.34 (t, J = 7.9 Hz, 1H, Ar–H), 7.15 (dd, J = 13.1, 4.3 Hz, 1H, Ar–H), 7.05–6.92 (m, 1H, Ar–H), 6.84–6.74 (m, 1H, Ar–H), 6.08 (d, J = 37.2 Hz, 1H, –N–CH–S), 4.69–4.51 (m, 1H, CH_2_–S), 3.94 (t, J = 7.2 Hz, 1H, CH_2_–S), 3.90–3.86 (s, 3H, CH_3_ ester), 3.78 (d, J = 3.1 Hz, 3H, OCH_3_), 3.66–3.56 (m, 1H, CH–COOCH_3_), 3.40 (dd, J = 69.6, 5.1 Hz, 2H, CH_2_ arg), 3.11 (ddd, J = 11.9, 9.6, 6.4 Hz, 1H, N–CH_2_), 2.72–2.53 (m, 1H, N–CH_2_), 2.46 (dt, J = 15.2, 6.2 Hz, 1H, CH_2_–CO), 2.01–1.86 (m, 1H, CH_2_–CO), 1.78–1.48 (m, 4H, 2CH_2_ arg); ^13^C-NMR (δ ppm): 172.98, 171.33, 164.37 (3C, CO), 159.94 (C_guanid_), 157.47, 130.82 (2C, C_Ar_), 127.38 (CH_Ar_), 121.55 (2C, CH_Ar_), 111.78 (CH_Ar_), 63.95 (S–CH–N), 56.21 (CH), 53.30 (OCH_3_), 40.81 (CH_2_), 39.19 (–CH_2_N–), 34.53 (–CH_2_S–), 33.10 (CH_2_), 31.43 (–CH_2_CO), 24.91 (CH_2_), 22.17 (CH_3_); RMS (EI-MS): *m*/*z* calculated for C_20_H_28_N_6_O_7_S [M + H]^+^ 497.1813; found 497.1814; Green chemistry metrics: E-factor 2.079, ME 0.325.

### *N*^2^-[(2-(3-Nitrophenyl)-4-oxo-1,3-thiazolidin-3-yl)propionyl]-nitro-l-arginine methyl ester (**6i**)

Light yellow cristals, mp 100 °C, yield: 50 %; IR (Zn/Se crystal, cm^−1^): 3302 (–NH); 3090, 783 (=C–H phenyl); 2951, 1257, 729 (–CH_2_–); 1736 (COOCH_3_); 1651 (–CONH); 1632 (C=O_thiazolidine-4-one_); 1601 (–C=C–_phenyl_); 1528, 1350 (NO_2_); 1219, 1095 (–C–N–); 683 (C–S); ^1^H-NMR (δ ppm): 8.58 (s, 1H, NH–CO), 8.21 (d, J = 4.3 Hz, 2H, Ar–H), 8.03 (m, 1H, NH), 7.77–7.67 (m, 1H, Ar–H), 7.61 (t, J = 8.0 Hz, 1H, Ar–H), 7.49–7.34 (m, 2H, NH), 5.93 (d, J = 34.7 Hz, 1H, –N–CH–S), 4.60 (s, 1H, CH_2_–S), 3.98–3.83 (m, 1H, CH_2_–S), 3.79 (s, 3H, CH_3_ ester), 3.76 (d, J = 4.6 Hz, 1H, CH–COOCH_3_), 3.59–3.39 (m, 2H, CH_2_ arg), 3.36–3.22 (m, 1H, N–CH_2_), 3.24–3.00 (m, 1H, N–CH_2_), 2.74–2.35 (m, 2H, CH_2_–CO), 1.84–1.55 (m, 4H, 2CH_2_ arg); ^13^C-NMR (δ ppm): 173.24, 171.67, 168.34 (3C, CO), 160.14 (C_guanid_), 149.41, 142.55 (2C, C_Ar_), 135.30, 133.92, 131.10, 129.30 (4C, CH_Ar_), 63.58 (S–CH–N), 53.54 (CH), 41.32 (CH_2_), 39.41 (CH_2_N–), 34.37 (–CH_2_S–), 33.48 (CH_2_), 31.94 (–CH_2_CO), 24.27 (CH_2_), 22.37 (CH_3_); HRMS (EI-MS): *m*/*z* calculated for C_19_H_25_N_7_O_8_S [M + H]^+^ 512.1558; found 512.1554; Green chemistry metrics: E-factor 3.687, ME 0.2134.

### *N*^2^-[(2-(2-Nitrophenyl)-4-oxo-1,3-thiazolidin-3-yl)propionyl]-nitro-l-arginine methyl ester (**6j**)

Yellow cristals, mp 95 °C, yield: 98 %; IR (Zn/Se crystal, cm^−1^): 3302 (–NH); 2983, 767 (=C–H_phenyl_); 2954, 1257, 725 (–CH_2_–); 1736 (COOCH_3_); 1659 (–CONH); 1628 (C=O_thiazolidine-4-one_); 1606 (–C=C–_phenyl_); 1524, 1342 (NO_2_); 1215, 1115 (–C–N–); 687 (C–S); ^1^H-NMR (δ ppm): 8.58 (s, 1H, NH–CO), 8.21 (m, 1H, NH), 8.15–8.06 (m, 1H, Ar–H), 7.72 (dd, J = 11.5, 3.8 Hz, 1H, Ar–H), 7.52 (dd, J = 11.4, 4.0 Hz, 1H, Ar–H), 7.35–7.28 (m, 1H, Ar–H), 7.20–7.14 (m, 2H, NH), 6.30 (d, J = 23.8 Hz, 1H, –N–CH–S), 4.57 (d, J = 7.2 Hz, 1H, CH_2_–S), 4.07–3.94 (m, 1H, CH_2_–S), 3.77 (d, J = 13.6 Hz, 3H, CH_3_ ester), 3.71–3.66 (m, 1H, CH–COOCH_3_), 3.61 (dd, J = 15.7, 2.8 Hz, 2H, CH_2_ arg), 3.49–3.28 (m, 1H, N–CH_2_), 3.17–3.02 (m, 1H, N–CH_2_), 2.71–2.47 (m, 1H, CH_2_–CO), 2.02–1.85 (m, 1H, CH_2_–CO), 1.77–1.61 (m, 4H, 2CH_2_ arg); ^13^C-NMR (δ ppm): 172.01, 170.56, 162.34 (3C, CO), 159.04 (C_guanid_), 146.93, 136.09 (2C, C_Ar_), 134.38, 129.13, 125.70, 116.24 (4C, CH_Ar_), 63.95 (S–CH–N), 58.72 (CH), 40.21 (CH_2_), 39.33 (–CH_2_N–), 33.64 (–CH_2_S–), 31.27 (CH_2_), 31.94 (–CH_2_CO), 29.55 (CH_2_), 24.26 (CH_3_); HRMS (EI-MS): *m*/*z* calculated for C_19_H_25_N_7_O_8_S [M + H]^+^ 512.1558; found 512.1559; Green chemistry metrics: E-factor 1.218, ME 0.452.

## Biological evaluation

### Antioxidant activity

#### DPPH radical scavenging assay

The radical scavenging activity of the tested compounds towards 1,1-diphenyl-2-picrylhydrazyl (DPPH) radical was measured as described in literature [32] with minor modifications. The samples were dissolved in DMSO in order to form the stock solutions with the concentration of 20 mg/mL. From the stock solutions there were taken different volumes (50, 100, 150, 200 µL) and completed up to 200 µL with methanol, then it was added 2800 µL of 0.1 mM DPPH methanol solution. The resulting mixture was kept in the dark for 60 min after which the absorbance was read at 517 nm against methanol, used as a blank solution. The final concentration of sample in the test tube was 0.33, 0.66, 0.99 and 1.32 mg/mL respectively. The DPPH radical-inhibiting capacity (radical scavenging ability) was calculated using the following formula:

1 $${\text{Inhibition}}\;\left( {{\text{scavenging}}\;{\text{activity}}} \right)\% = \left[ {\left( {{\text{A}}_{\text{C}} - {\text{ A}}_{\text{S}} } \right)/{\text{A}}_{\text{C}} } \right] \times 100$$

where A_C_ = absorbance of the DPPH solution, A_S_ = absorbance of the sample. Vitamin E (α-tocopherol) was used as positive control and as references were used NO_2_-Arg-OMe and l-arginine, all three being processed in a similar manner with the samples. All determinations were performed in triplicate.

#### ABTS radical scavenging assay

The generation of radical cation ABTS^·+^ was carried out by treating the aqueous solution of 2,2′-azino-bis (3-ethylbenzothiazoline-6-sulfonic acid) (7 mM) with ammonium persulfate (2.45 mM). The resulting mixture was kept in the dark for 16 h to promote the formation of ABTS^·+^, as described in [33, 34]. The ABTS^+^ radical cation solution was diluted with ethanol to obtain an absorbance value of 0.7 ± 0.02 at 734 nm. Different sample volumes (10, 15, 25, 50 µL) from a stock solution of 20 mg/mL in DMSO were mixed with DMSO to 50 µL and then 1950 µL of ABTS^·+^ solution were added. The final concentration of sample in the test tube was 0.1, 0.15, 0.25 and 0.50 mg/mL respectively. After 6 min the absorbance was measured at 734 nm against a blank (ethanol) and the radical scavenging capacity was calculated according to the following equation:

2 $${\text{Scavenging}}\;{\text{activity}}\;\% = \left[ {\left( {{\text{A}}_{\text{C}} {-}{\text{A}}_{\text{S}} } \right)/{\text{A}}_{\text{C}} } \right] \times 100$$

where A_C_ = absorbance of ABTS^·+^ alcoholic solution; A_S_ = absorbance of the samples, read at 6 min after the addition of the ABTS^·+^ solution. Vitamin E (α-tocopherol) was used as positive control and as references were used NO_2_-Arg-OMe and l-arginine, all three being processed in a similar manner with the samples. All determinations were performed in triplicate.

#### Phosphomolydenum reducing antioxidant power (PRAP) assay

The total antioxidant activity of tested compounds was evaluated using the phosphomolybdenum method according to the procedure described in the literature [35] with minor modifications. For each compound was prepared a stock solution with the concentration of 20 mg/mL in DMSO, from which there were used different volumes (20, 40, 60, 80 µL) and completed with DMSO up to 200 µL. Over these samples it was added 2 mL of the reagent solution (0.6 M sulfuric acid, 28 mM disodium hydrogen phosphate, and 4 mM ammonium molybdate). The samples were incubated at 95 °C for 90 min at drying stove (oven). The final concentration of sample in the test tube was 0.18, 0.36, 0.54 and 0.72 mg/mL respectively. After cooling to room temperature, the absorbance was read at 695 nm against a blank (200 mL DMSO + 2 mL reagent). Vitamin E (α-tocopherol) was used as positive control and as references were used NO_2_-Arg-OMe and l-arginine, all three being processed in a similar manner with the samples. All determinations were performed in triplicate.

#### Ferric reducing antioxidant power (FRAP) assay

The ferric reducing antioxidant power of the compounds was quantified by the method described by [36] with slight modifications. The compounds were tested at different concentrations (20, 10, 5, 2.5 mg/mL). To 0.5 mL of samples of each concentration it was added 0.5 mL of 0.2 M phosphate buffer pH 6.6. The reaction was then initiated by the addition of 0.5 mL of potassium ferricyanide 1 % w/v, after which the samples are incubated at 50 °C (oven) for 20 min and the completion of the reaction takes place by addition of 0.5 mL trichloroacetic acid 10 % w/v. 1 mL from the resulting solution of each sample was diluted with 1 mL double distilled deionised water and finally 0.2 mL of ferric chloride 0.1 % w/v was added. The final concentration of sample in the test tube was 4.5454, 2.2727, 1.1360, 0.5681 mg/mL respectively. The mixture was left at room temperature for 10 min and then the absorbance was measured at 700 nm against a blank solution prepared similar to the sample, which contain 0.5 mL DMSO instead 0.5 mL sample. Vitamin E (α-tocopherol) was used as positive control and as references were used NO_2_-Arg-OMe and l-arginine, all three being processed in a similar manner with the samples. All determinations were performed in triplicate.

#### Antibacterial/antifungal assays

##### Agar disc diffusion method

Antibacterial and antifungal activity of the **6a**–**j** derivatives expressed as diameter of inhibition area was evaluated by the standard disk diffusion assay according to described protocols [42]. Prior to use, the strains (bacteria and yeasts) were diluted in sterile 0.9 % NaCl until the turbidity was equivalent to McFarland standard no. 0.5 (106 CFU/mL). The suspensions were further diluted 1:10 in Mueller–Hinton agar for bacteria and Sabouraud agar for fungi and then spread on sterile Petri plates (25 mL/Petri plate). Sterile stainless steel cylinders (5 mm internal diameter; 10 mm height) were applied on the agar surface in Petri plates. In each cylinder 200 μL of sample solutions in DMSO (20 mg/mL) was added. As positive control there were used commercial available discs containing ampicillin (25 mcg/disc), chloramphenicol (30 mcg/disc) and nystatin (100 mcg/disc). DMSO was used as a negative control. The plates were incubated at 37 °C for 24 h (bacteria) and at 24 °C for 48 h (fungi). The diameters of inhibition area developed after the incubation were measured.

##### The broth micro-dilution method

The minimum inhibitory concentration (MIC) and the minimum bactericidal/fungicidal concentration (MBC/MFC) against bacteria and fungi respectively were determined by the two-fold dilution method, with minor modification [38]. The active cultures of the bacteria and fungi were prepared by transferring the loopful of cells from the stock culture to the conical flasks containing Mueller–Hinton broth for bacteria or Sabouraud broth for fungi. The cultures were incubated at 37 °C for 24 h (bacteria) and at 24 °C for 48 h (fungi) and then were diluted with fresh media to obtain an optical density value of 106 CFU/mL. Different dilutions of the **6a**–**j** derivatives made in the Mueller–Hinton broth (bacteria) and in Sabourand broth (fungi) were prepared in a 96-well microplate by the twofold dilution method in the concentration range of 10, 5, 2.5, 1.25, 0.625, 0.312, 0.156, 0.078, 0.039, 0.0195, 0.009 and 0.0048 mg/mL. Then 10 µL of each strain (106 CFU/mL) was inoculated onto the microplates. The plates were incubated again at 37 °C for 24 h. The lowest concentrations of the tested compounds which did not show any visual growth of the test strain, were determined as the MICs, which were expressed in mg/mL. For the determination of MBCs and MFCs, the MIC and the next higher concentrations of the sample were selected, spread on the agar plates, and incubated at 37 °C for 24 h. The concentration of the tested compounds, which did not show any growth of the microorganism on the agar plates, was determined as the MBC/MFC and expressed in mg/mL. Each determination was performed in triplicate.

## Conclusions

The present work is centered on the synthesis and biological evaluation of new thiazolidine-4-ones derived from the methyl ester of nitro-l-arginine. The structure of the compounds was proven using spectral methods (IR, ^1^H-NMR, ^13^C-NMR, MS). The antioxidant activity was quantified using four in vitro tests: DPPH/ABTS scavenging assays and ferric/phosphomolybdenum reducing antioxidant power assays. The *methoxy*-substituted derivatives, **6h** (R = 2-OCH_3_) and **6g** (3-OCH_3_), showed a high free radical scavenging ability, both for DPPH and ABTS radicals. A good influence was exerted also by the *nitro* and *bromo* substitution. The 2-*nitro*-derivative, **6j**, showed the best ABTS scavenging ability while the 4-*bromo*-derivative, **6e**, presented the best ferric and phosphomolybdenum reducing antioxidant power. The compound **6j** also showed a good antibacterial and antifungal activity. It was the most active on *S. aureus*, *S. lutea* and *P. aeruginosa* and *Candida* spp. respectively. The encouraging preliminary results support the antioxidant and antibacterial/antifungal potential of the synthesized compounds and their possible applications in several diseases mediated by reactive oxygen species (ROS) and susceptible to infections such as wound healing from burns.
